# Supplementary material for: SARS-CoV-2 mRNA vaccination exposes progressive adaptive immune dysfunction in patients with chronic lymphocytic leukemia
Source: medRxiv. 2022 Dec 20:2022.12.19.22283645. Preprint. [Version 1] doi: 10.1101/2022.12.19.22283645 (PMC9810225; doi:10.1101/2022.12.19.22283645)
Supplement: 1 [file NIHPP2022.12.19.22283645V1-supplement-1.pdf]

Figure S1

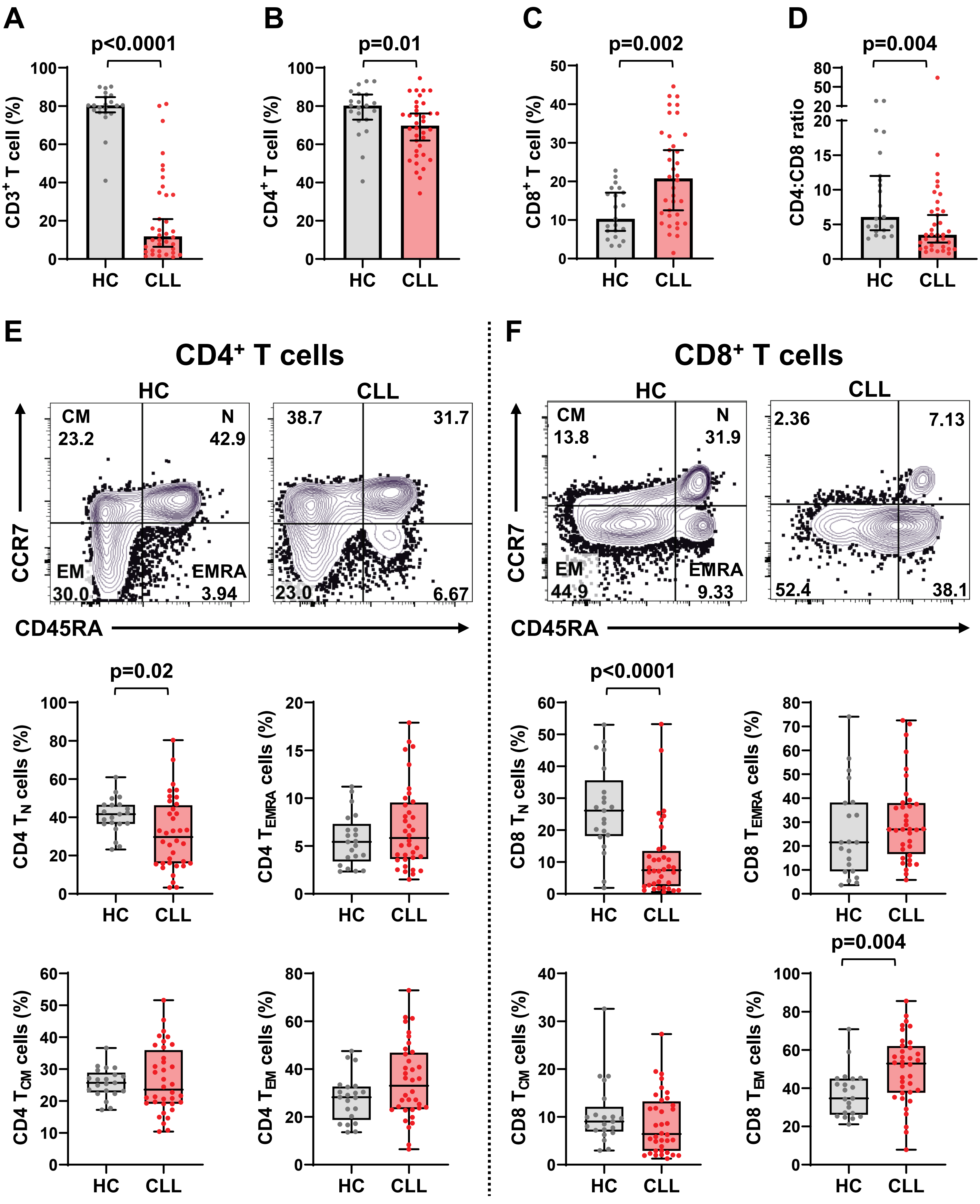

A
p<0.0001
CD3+ T cell (%)
HC
CLL
B
p=0.01
CD4+ T cell (%)
C
p=0.002
CD8+ T cell (%)
D
p=0.004
CD4:CD8 ratio
E
CD4+ T cells
HC
CLL
CM
23.2
N
42.9
EM
30.0
EMRA
3.94
38.7
31.7
23.0
6.67
CCR7
CD45RA
F
CD8+ T cells
CM
13.8
N
31.9
EM
44.9
EMRA
9.33
2.36
7.13
52.4
38.1
p=0.02
CD4 TN cells (%)
CD4 TEMRA cells (%)
p<0.0001
CD8 TN cells (%)
CD8 TEMRA cells (%)
CD4 TCM cells (%)
CD4 TEM cells (%)
CD8 TCM cells (%)
p=0.004
CD8 TEM cells (%)

Figure S1. CLL patients have altered total $CD4^+$ and $CD8^+$ T cell and subpopulation frequencies, Related to Figures 2 & 5.

Immunophenotyping of PBMCs from vaccinated HC (n=21) and CLL (n=36) donors.

(A-D) Quantitative comparisons of total $CD3^+$ (A), $CD4^+$ (B), and $CD8^+$ (C) T cell frequencies and CD4:CD8 ratios (D).

(E-F) Representative flow cytometry plots and quantitative comparisons of naïve (N), central memory (CM), effector memory (EM) and effector memory $CD45RA^+$ (EMRA) $CD4^+$ (E) and $CD8^+$ (F) subpopulation frequencies defined by the CCR7 and CD45RA surface markers in HC and CLL donors.

Bars indicate the median with 95% CI. P values were determined by the Mann-Whitney test.

Figure S2

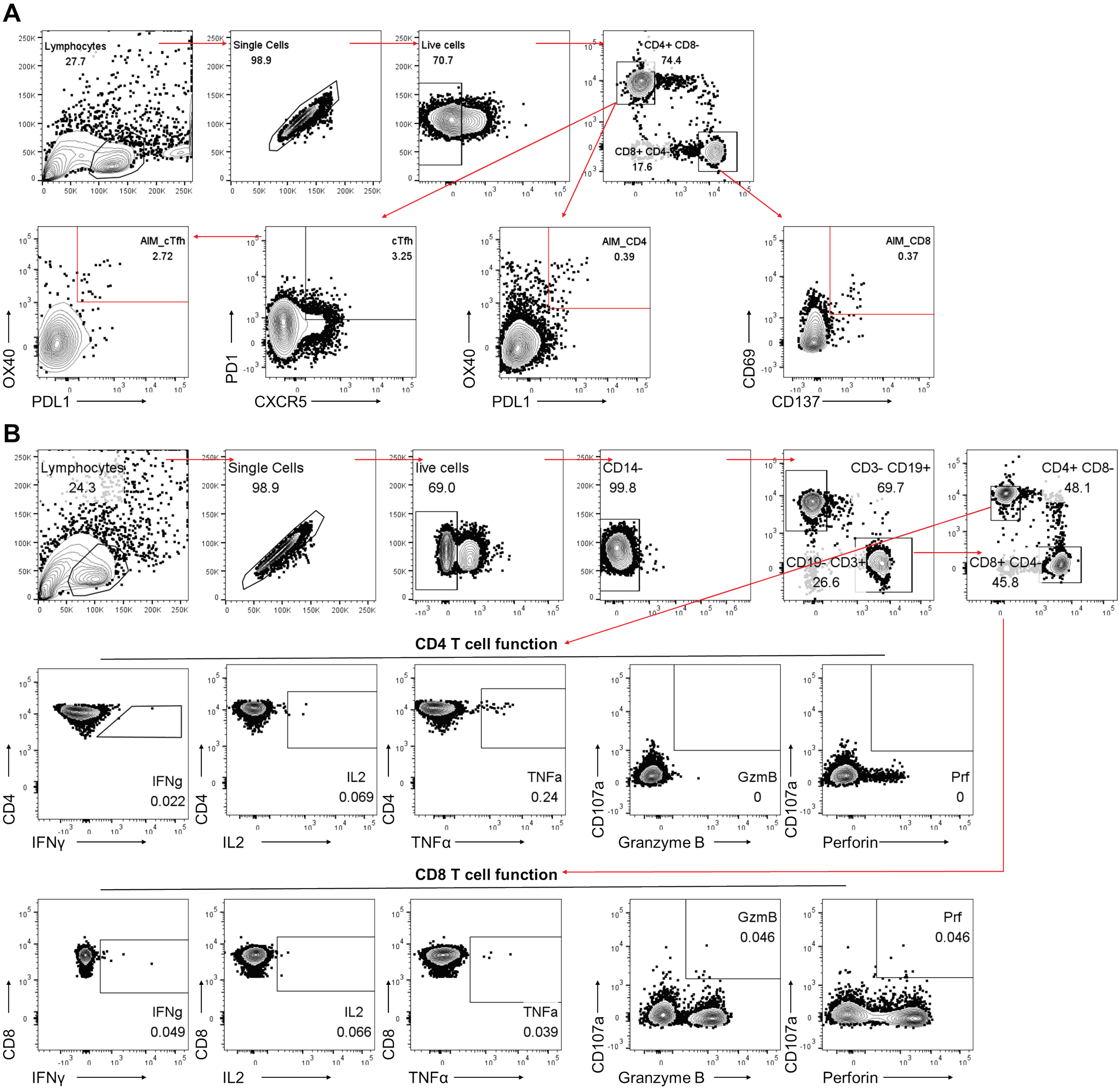

Figure S2. Flow cytometry gating strategies for measuring T cell responses from a CLL donor after S peptide pool stimulation, Related to Figure 2.

(A) Gating strategy to examine activation-induced markers (AIM) by CD4$^+$, cTfh, and CD8$^+$ T cells.

(B) Gating strategy to examine CD4$^+$ and CD8$^+$ T cell effector function by intra-cellular staining (ICS).

Figure S3

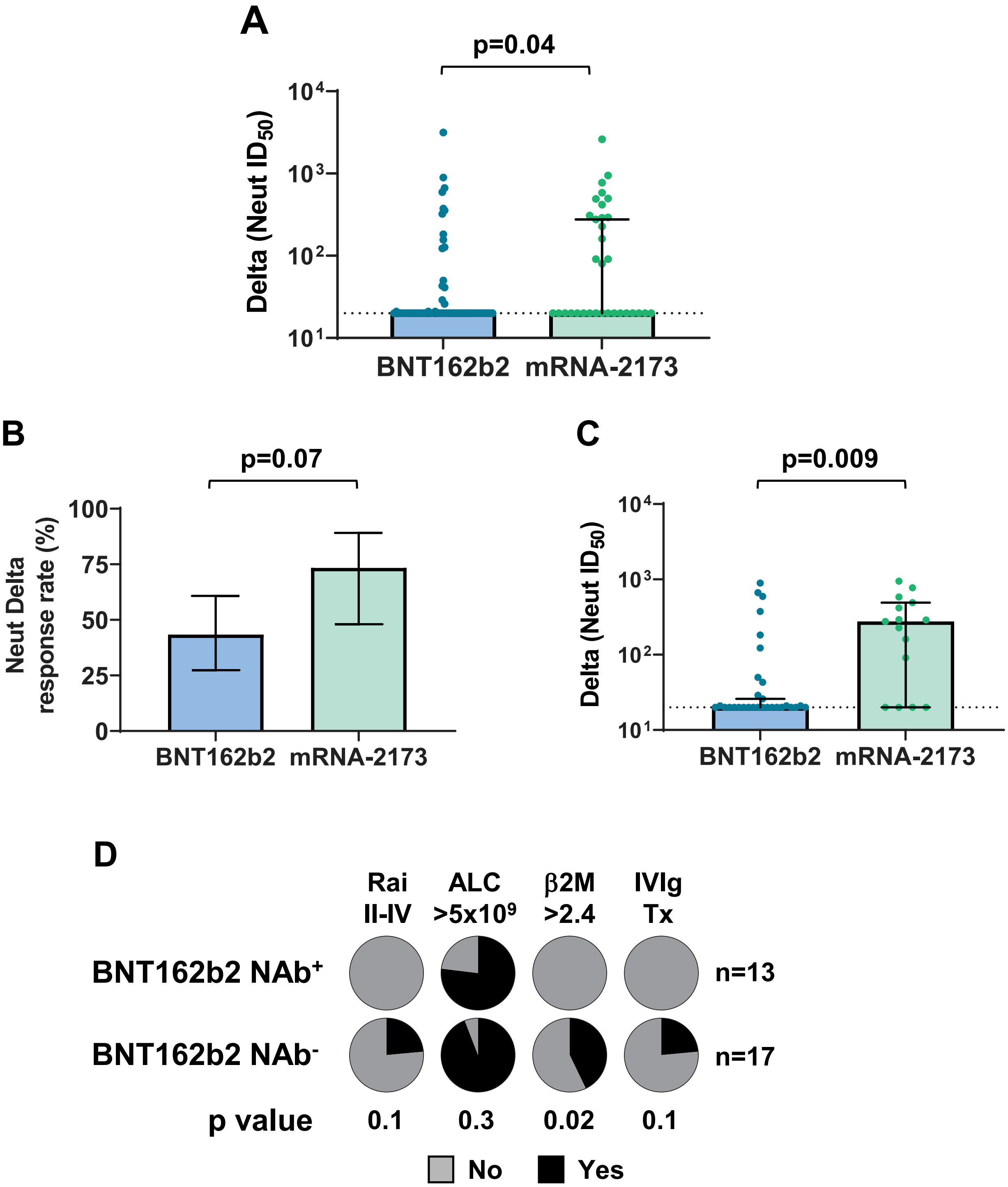

A
p=0.04
Delta (Neut ID50)
10⁴
10³
10²
10¹
BNT162b2
mRNA-2173
B
p=0.07
Neut Delta response rate (%)
100
75
50
25
0
BNT162b2
mRNA-2173
C
p=0.009
Delta (Neut ID50)
10⁴
10³
10²
10¹
BNT162b2
mRNA-2173
D
Rai II-IV
ALC >5x10⁹
β2M >2.4
IVIg Tx
BNT162b2 NAb⁺
n=13
BNT162b2 NAb⁻
n=17
p value
0.1
0.3
0.02
0.1
No
Yes

Figure S3. NAb titers against the Delta variant are higher in total and treatment-naïve mRNA-2173 CLL vaccinees, but lower in patients with clinical progression, Related to Figure 4.

(A) Comparison of ID_{50} neutralizing titers against the SARS-CoV-2 Delta variant for CLL patients classified by BNT162b2 (n=60) or mRNA-2173 (n=33) vaccine type.

(B) Response rates for the generation of Delta NAbs by vaccine type in treatment-naive CLL patients.

(C) Comparison of ID_{50} titers against Delta for treatment-naive CLL patients by BNT162b2 (n=30) or mRNA-2173 (n=15) vaccine type.

(D) Frequencies of four clinical features between treatment-naive BNT162b2 vaccinees stratified by Delta NAb^{+} (n=13) or NAb^{-} (n=17) serologic status.

Bars indicate the median with 95% CI (A and C) or mean (B). P values were calculated with the Mann-Whitney test (A and C) or Fisher's exact test (B and D).

Table S1. Disease characteristics and features of SARS-CoV-2 vaccinated CLL subjects, Related to Figures 1 & 4 and Tables 1 & S4.

		Clinical Features			Prognostic Markers		Cytogenetics by FISH					Therapeutic History and Type						Humoral Immune Status				Vaccine Features	
													Anti-CD20 Tx		BTKi Tx			Ig Isotypes (mg/L)					
	CLL Sample	Rai Stage Naïve or Off Tx R/R (II-IV=1)	ALC (>5,000 [10^9/L] =1)	β2M (≤2.4 mg/L=1)	*IGHV* (MT=1)	CD38 (>20%=1)	13q	tri12	WNL	11q	17p	Prior Treatment (Y=1)	<12M	≥12M	Alone	Combo[a]	Other Active Tx	IgA (≥60=1)	IgG (≥650=1)	IgM (≥40=1)	IVIg Tx	Type (Pfizer=1; Moderna=0)	Months from #2 VAX (≤1/2/≥3)
1	CLLV1		0	0	0	0	1					1	1			1					1	1	1
2	CLLV2	0	1	1	0	1		1				0						1	0	0	0	1	1
3	CLLV3		0	1	0	0	1					1		1	1			1	0	0	0	1	1
4	CLLV4		0	1	0	0				1		1		1							1	0	1
5	CLLV5		0	0	ND	1					1	1		1		1	I/BTKi				1	0	1
6	CLLV6		1	1	ND	0	1					1			1						1	1	2
7	CLLV7		0	0	ND	1		1				1		1		1	P/BTKi				1	1	2
8	CLLV8	0	1	1	1	0	1					0									1	0	1
9	CLLV9		0	ND	1	0	1					1		1							1	1	1
10	CLLV10	0	1	1	ND	1	1					0						1	1	1	0	0	1
11	CLLV11		0	0	0	1		1				1	1					1	1	0	0	1	1
12	CLLV12	1	1	ND	1	0	1					0									1	1	1
13	CLLV13		1	1	0	1				1		1		1	1			0	0	0	0	1	2
14	CLLV14		1	1	0	0		1				1		1			P	1	1	0	0	1	1
15	CLLV15	0	1	ND	1	0	1					0									1	0	1
16	CLLV16		0	1	ND	ND		1				1	1			1		1	1	1	0	0	1
17	CLLV17		0	0	0	1	ND	ND	ND	ND	ND	1		1	1						1	0	1
18	CLLV18		0	1	1	0	1					1	1			1	A/BTKi/V	1	1	0	0	1	2
19	CLLV19	0	1	0	1	0	ND	ND	ND	ND	ND	0						0	0	0	0	0	1
20	CLLV20	0	1	1	1	1	1					0						1	0	0	0	1	2
21	CLLV21	1	1	0	1	1			1			0						1	0	0	0	1	2
22	CLLV22	0	0	1	0	0	1					1	1								1	1	2
23	CLLV23	0	1	1	1	0			1			0						1	1	1	0	1	2
24	CLLV24		0	1	0	0			1			1	1					1	1	1	0	0	1
25	CLLV25		0	0	ND	0	1					1		1			C	0	0	1	0	1	2
26	CLLV26		0	1	ND	0		1				1		1	1			0	0	0	0	0	1
27	CLLV27	0	1	1	1	1	1					0						1	1	1	0	1	2
28	CLLV28	0	1	ND	1	0			1			0						1	0	0	0	0	2
29	CLLV29		0	0	0	0	1					1			1			1	0	0	0	1	2
30	CLLV30	1	1	1	1	0					1	0						1	1	0	0	0	2
31	CLLV31	0	1	0	1	0	1					1									1	1	1
32	CLLV32	0	0	1	1	0	1					0						1	1	0	0	1	3
33	CLLV33	0	0	1	1	0	1					0						1	1	1	0	1	3
34	CLLV34	0	1	0	1	0			1			0						1	1	0	0	1	1
35	CLLV35		0	1	ND	0		1				1		1				1	1	1	0	1	1
36	CLLV36	0	1	1	1	0	1					0									1	1	2
37	CLLV37		0	0	0	0					1	1		1				1	1	1	0	1	1
38	CLLV38	0	1	1	1	0					1	0						1	1	1	0	1	2
39	CLLV39		0	1	1	0	1					1	1			1		0	0	0	0	1	2
40	CLLV40	1	1	1	1	0	1					1		1				0	0	0	0	1	2
41	CLLV41		0	0	0	1					1	1	1								1	0	1
42	CLLV42		1	1	0	0				1		1		1	1						1	1	2
43	CLLV43		0	1	ND	0	1					1			1			1	1	1	0	1	2
44	CLLV44	0	0	1	ND	ND	ND	ND	ND	ND	ND	0						1	1	0	0	1	2
45	CLLV45		0	0	0	0	1					1	1				A/PI3Ki/V	1	0	0	0	0	2
46	CLLV46	0	1	1	1	0	1					0						0	0	0	0	1	1
47	CLLV47		0	1	ND	ND		1				1		1				1	1	1	0	1	2
48	CLLV48	0	1	0	1	0	1					0						1	1	1	0	0	3
49	CLLV49	0	1	1	0	1				1		1		1				1	0	1	0	0	1
50	CLLV50		1	ND	1	0	1					1	1								1	1	2
51	CLLV51		0	0	0	1	1					1	1					0	0	0	0	0	1
52	CLLV52	0	1	1	1	0	1					0						1	1	0	0	1	3
53	CLLV53		0	1	ND	1	ND	ND	ND	ND	ND	1		1							1	1	3
54	CLLV54	0	1	0	1	0	1					1		1							1	0	2
55	CLLV55	1	1	ND	0	1			1			0						0	0	0	0	1	1
56	CLLV56	0	0	1	ND	0	1					0						1	1	1	0	0	3
57	CLLV57	0	1	1	1	0	1					0						1	0	0	0	1	2
58	CLLV58	0	1	1	1	0			1			0						0	1	0	0	1	1
59	CLLV59	0	1	ND	1	0					1	1						1	0	0	0	1	1
60	CLLV60	1	1	0	1	0		1				0									1	1	3
61	CLLV61		0	0	ND	0				1		1			1			1	1	1	0	1	3
62	CLLV62	0	1	1	1	0			1			0						1	1	0	0	0	3
63	CLLV63	0	0	0	ND	1	ND	ND	ND	ND	ND	0						0	1	0	0	0	3
64	CLLV64		0	1	0	1	1					1	1			1		1	1	1	0	1	1
65	CLLV65	0	1	1	1	0	1					0						1	0	0	0	1	3
66	CLLV66		0	0	0	1		1				1	1			1					1	0	3
67	CLLV67		0	ND	1	0	1					1		1				0	1	1	0	1	3
68	CLLV68	0	1	0	0	0				1		0						1	1	0	0	1	3
69	CLLV69	0	1	1	1	0	1					0						0	1	0	0	0	3
70	CLLV70		1	0	1	0	1					1					P	1	1	0	0	1	3
71	CLLV71		0	1	0	0				1		1		1			V				1	0	3
72	CLLV72		0	1	ND	0			1			1		1				1	1	1	0	0	3
73	CLLV73		0	0	0	0		1				1		1	1						1	1	3
74	CLLV74		0	ND	1	0					1	1				1	BTKi/V				1	0	3
75	CLLV75		1	ND	1	0	1					1	1					0	0	0	0	0	3
76	CLLV76	0	1	1	1	0	1					0						1	1	1	0	0	3
77	CLLV77		0	0	0	1				1		1		1			L				1	0	3
78	CLLV78	0	1	0	1	ND	1					0						1	1	0	0	1	3
79	CLLV79	0	1	ND	1	0	1					0						1	1	0	0	1	3
80	CLLV80	0	1	0	0	0	1					1		1				1	0	0	0	0	2
81	CLLV81		0	0	0	0				1		1		1	1			1	0	0	0	1	3
82	CLLV82	0	1	1	1	0	1					0						1	1	0	0	0	3
83	CLLV83		0	ND	ND	0			1			1	1			1		1	0	0	0	0	2
84	CLLV84	0	1	1	1	1	1					0						1	1	1	0	1	2
85	CLLV85	0	1	1	1	ND	1					0						1	1	0	0	1	2
86	CLLV86	0	1	1	1	1		1				0						1	0	1	0	1	3
87	CLLV87	0	1	0	1	0		1				0									1	1	3
88	CLLV88	0	1	1	0	1				1		0						1	1	1	0	1	2
89	CLLV89		0	ND	ND	0	1					1		1				1	1	1	0	1	3
90	CLLV90	0	1	1	1	0	1					0						1	0	1	0	1	3
91	CLLV91	0	0	1	ND	0		1				0						1	1	0	0	1	3
92	CLLV92		0	0	0	0		1				1			1			1	0	0	0	1	3
93	CLLV93	0	1	1	1	0	1					0						1	1	0	0	0	3
94	CLLV94	0	1	0	1	1	1					0						1	1	0	0	0	3
95	CLLV95	0	1	1	ND	0	1					0						1	0	0	0	1	3

[a]Combo indicates concomitant treatment with anti-CD20/BTKi unless otherwise specified in the Other Tx column

Values of "1" indicate the presence and "0" the absence of a variable. For values left blank, data are not applicable.

Abbreviations: R/R, relapsed refractory; ALC, absolute lymphocyte count; β2M, Beta 2 microglobulin; ND, no data; IGHV, immunoglobulin heavy chain variable region gene; MT, mutated; FISH, fluorescence in situ hybridization; WNL, within normal limits; Tx, treatment; BTKi, Bruton's tyrosine kinase inhibitor; I, imatinib; P, prednisone; A, anti-CD20; V, venetoclax; C, cyclosporine; PI3Ki, Phosphoinositide 3-kinase inhibitor; L, lenalidomide; IVIg, intravenous immunoglobulin; Pfizer-BioNTech, BNT162b2; Moderna, mRNA-2173

Table S2. Binding and neutralizing antibody titers in the plasma of SARS-CoV-2 vaccinated CLL patients and healthy controls, Related to Figures 1, 3 & 4.

	ID	Status	Gender	Binding antibodies									Neutralization	
				Spike		RBD		S1		S2		RBD/ACE2 binding	D614G	Delta
				EC_{50}	Endpoint	EC_{50}	Endpoint	EC_{50}	Endpoint	EC_{50}	Endpoint		ID_{50}	ID_{50}
1	HC1	Healthy	Male	3454	66220	1829	14885	2754	46347	1492	13180	54.2	237	171
2	HC2	Healthy	Female	44369	>312500	15247	239875	24253	>312500	10506	100315	0.9	3114	651
3	HC3	Healthy	Female	48276	>312500	20806	>312500	38100	>312500	14550	207896	11.7	3493	2711
4	HC4	Healthy	Male	9754	94317	2396	23760	3473	64473	4366	74455	20.5	230	125
5	HC5	Healthy	Male	5780	76936	2351	25047	4339	61521	2355	22565	19.9	897	319
6	HC6	Healthy	Female	9103	132297	2390	25922	4722	84416	3407	61522	17.6	1220	1959
7	HC7	Healthy	Female	23979	>312500	8471	103877	12452	135787	4468	69473	3.5	1263	1272
8	HC8	Healthy	Male	2247	26478	230	11830	1119	15479	448	14123	48.6	83	<20
9	HC9	Healthy	Female	108316	>312500	26151	>312500	54808	>312500	6334	132888	0.6	6398	2661
10	HC10	Healthy	Female	10050	151027	2527	31974	4976	110597	448	13278	20.3	398	77
11	HC11	Healthy	Male	13974	177209	3377	53091	4510	82832	3048	54050	18.5	1046	339
12	HC12	Healthy	Female	15911	250229	2439	26567	5186	61195	2018	19229	28.7	423	147
13	HC13	Healthy	Male	15433	266151	4678	112835	10706	130281	4662	76938	24.9	1437	511
14	HC14	Healthy	Female	16199	262987	4069	86869	7589	107134	2802	46163	5.5	2156	1390
15	HC15	Healthy	Female	34574	>312500	12719	201615	ND	ND	ND	ND	1.9	471	479
16	HC16	Healthy	Female	37503	>312500	16553	184510	ND	ND	ND	ND	4.6	1045	1288
17	HC17	Healthy	Female	18933	>312500	5864	91555	ND	ND	ND	ND	2.2	427	353
18	HC18	Healthy	Male	15276	225339	6343	87278	ND	ND	ND	ND	8.7	691	533
19	HC20	Healthy	Male	28325	>312500	9890	120823	ND	ND	ND	ND	3.5	964	709
20	HC21	Healthy	Male	37411	>312500	12777	160705	ND	ND	ND	ND	4.6	1221	677
21	HC22	Healthy	Male	9945	80034	3040	44720	ND	ND	ND	ND	14.4	442	320
22	HC23	Healthy	Female	8790	127507	2648	34505	ND	ND	ND	ND	22.3	280	251
23	HC24	Healthy	Male	46399	>312500	27919	>312500	ND	ND	ND	ND	11.6	1528	1295
24	HC25	Healthy	Male	15586	225216	5616	89684	ND	ND	ND	ND	13.5	368	310
25	HC26	Healthy	Male	2712	45027	791	15080	ND	ND	ND	ND	61.8	42	39
26	HC27	Healthy	Female	10379	94070	1439	17079	ND	ND	ND	ND	52.3	114	71
27	HC28	Healthy	Male	42858	>312500	3682	73382	8005	128500	11187	116170	3.4	457	514
28	HC29	Healthy	Female	2230	27529	763	13108	ND	ND	ND	ND	72	51	53
29	HC30	Healthy	Male	4425	96677	1669	13452	ND	ND	ND	ND	49	85	140
30	HC31	Healthy	Male	1480	15125	402	7702	ND	ND	ND	ND	64.5	<20	<20
1	CLLV1	On.Tx	Male	<100	<100	<100	<100	ND	ND	ND	ND	>90	<20	<20
2	CLLV2	Tx.naïve	Male	10814	116098	2886	38114	ND	ND	ND	ND	49.8	308	595
3	CLLV3	On.Tx	Female	438	13445	159	559	267	2115	228	6362	>90	<20	<20
4	CLLV4	Off.Tx.CR	Female	2097	19504	433	10349	ND	ND	ND	ND	59.9	59	91
5	CLLV5	On.Tx	Female	<100	3461	<100	<100	<100	154	179	5001	>90	<20	<20
6	CLLV6	On.Tx	Female	<100	<100	<100	<100	ND	ND	ND	ND	>90	<20	<20
7	CLLV7	On.Tx	Female	<100	<100	<100	<100	ND	ND	ND	ND	>90	<20	<20
8	CLLV8	Tx.naïve	Female	37157	>312500	10300	142185	ND	ND	ND	ND	11.5	2371	946
9	CLLV9	Off.Tx.CR	Female	870	16255	247	5147	ND	ND	ND	ND	82.8	38	41
10	CLLV10	Tx.naïve	Male	18335	262114	5265	75564	ND	ND	ND	ND	11.1	840	419
11	CLLV11	On.Tx	Male	<100	<100	<100	<100	ND	ND	ND	ND	>90	<20	<20
12	CLLV12	Tx.naïve	Male	<100	533	<100	323	ND	ND	ND	ND	>90	<20	<20
13	CLLV13	On.Tx	Female	<100	<100	<100	<100	ND	ND	ND	ND	>90	<20	<20
14	CLLV14	On.Tx	Male	<100	<100	<100	<100	ND	ND	ND	ND	>90	<20	<20
15	CLLV15	Tx.naïve	Female	<100	<100	<100	<100	ND	ND	ND	ND	>90	<20	<20
16	CLLV16	On.Tx	Male	352	6938	<100	<100	200	2552	395	11303	>90	<20	<20
17	CLLV17	On.Tx	Female	<100	<100	<100	<100	ND	ND	ND	ND	>90	<20	<20
18	CLLV18	On.Tx	Female	<100	<100	<100	<100	ND	ND	ND	ND	>90	<20	<20
19	CLLV19	Tx.naïve	Female	42825	>312500	15085	201882	ND	ND	ND	ND	4.1	1857	490
20	CLLV20	Tx.naïve	Male	184	6770	<100	<100	143	4032	<100	<100	>90	<20	<20
21	CLLV21	Tx.naïve	Male	13018	164471	<100	<100	<100	<100	11869	161466	>90	<20	<20
22	CLLV22	Off.Tx.and.R/R	Male	2022	13899	<100	<100	<100	<100	6829	110325	>90	<20	<20
23	CLLV23	Tx.naïve	Female	3170	37209	608	12919	ND	ND	ND	ND	85.7	29	26
24	CLLV24	On.Tx	Male	<100	<100	<100	<100	ND	ND	ND	ND	>90	<20	<20
25	CLLV25	On.Tx	Female	<100	<100	<100	<100	ND	ND	ND	ND	>90	<20	<20
26	CLLV26	On.Tx	Male	10869	148175	<100	<100	1986	12518	23339	>312500	>90	<20	<20
27	CLLV27	Tx.naïve	Female	166	6293	<100	<100	ND	ND	ND	ND	53.2	314	896
28	CLLV28	Tx.naïve	Male	2942	51890	383	10082	ND	ND	ND	ND	62.2	153	276
29	CLLV29	On.Tx	Male	<100	<100	<100	<100	ND	ND	ND	ND	>90	<20	<20
30	CLLV30	Tx.naïve	Male	2913	43861	1809	11362	ND	ND	ND	ND	65.9	265	91
31	CLLV31	Off.Tx.and.R/R	Female	<100	<100	<100	<100	ND	ND	ND	ND	>90	<20	<20
32	CLLV32	Tx.naïve	Female	731	15965	123	1405	ND	ND	ND	ND	>90	22	21
33	CLLV33	Tx.naïve	Male	254	11569	253	1260	ND	ND	ND	ND	>90	21	<20
34	CLLV34	Tx.naïve	Female	790	15389	169	1206	142	5117	864	19779	>90	<20	<20
35	CLLV35	Off.Tx.CR	Female	342	9254	171	2378	ND	ND	ND	ND	>90	22	<20
36	CLLV36	Tx.naïve	Female	647	13147	198	4074	ND	ND	ND	ND	>90	<20	<20
37	CLLV37	Off.Tx.CR	Male	31919	>312500	14039	188186	ND	ND	ND	ND	4	2095	3145
38	CLLV38	Tx.naïve	Male	5424	89373	1770	20417	ND	ND	ND	ND	40.5	273	665
39	CLLV39	On.Tx	Male	<100	<100	<100	<100	ND	ND	ND	ND	>90	<20	<20
40	CLLV40	Off.Tx.and.R/R	Female	<100	<100	<100	<100	ND	ND	ND	ND	>90	<20	<20
41	CLLV41	On.Tx	Male	<100	<100	<100	<100	ND	ND	ND	ND	>90	<20	<20
42	CLLV42	On.Tx	Female	<100	<100	<100	<100	ND	ND	ND	ND	>90	<20	<20
43	CLLV43	On.Tx	Female	1302	21707	<100	<100	<100	1274	3491	58888	>90	<20	NA
44	CLLV44	Tx.naïve	Female	3175	46365	549	12788	ND	ND	ND	ND	>90	247	43
45	CLLV45	On.Tx	Female	<100	<100	<100	<100	ND	ND	ND	ND	>90	<20	NA
46	CLLV46	Tx.naïve	Male	662	12679	<100	<100	<100	1784	3018	45157	>90	<20	<20
47	CLLV47	Off.Tx.CR	Female	19885	303589	3338	60781	ND	ND	ND	ND	22.9	508	355
48	CLLV48	Tx.naïve	Female	258	4612	<100	1134	ND	ND	ND	ND	>90	<20	<20
49	CLLV49	Off.Tx.and.R/R	Male	4335	80421	144	4204	ND	ND	ND	ND	>90	69	308
50	CLLV50	On.Tx	Female	<100	<100	<100	<100	ND	ND	ND	ND	>90	<20	<20
51	CLLV51	On.Tx	Male	<100	<100	<100	<100	ND	ND	ND	ND	>90	<20	<20
52	CLLV52	Tx.naïve	Female	3800	66371	1706	14991	ND	ND	ND	ND	45.3	85	50
53	CLLV53	Off.Tx.CR	Male	2740	29793	708	16666	ND	ND	ND	ND	58.7	65	156
54	CLLV54	Off.Tx.and.R/R	Female	<100	<100	<100	<100	ND	ND	ND	ND	>90	<20	<20
55	CLLV55	Tx.naïve	Male	<100	1672	<100	<100	<100	<100	<100	1841	>90	<20	<20
56	CLLV56	Tx.naïve	Male	2616	51938	1172	19650	ND	ND	ND	ND	31.1	240	293
57	CLLV57	Tx.naïve	Female	6062	141781	1185	16644	ND	ND	ND	ND	40.8	407	123
58	CLLV58	Tx.naïve	Female	4564	76566	228	7036	ND	ND	ND	ND	>90	24	21
59	CLLV59	Off.Tx.and.R/R	Male	305	11023	166	3255	ND	ND	ND	ND	64	667	127
60	CLLV60	Tx.naïve	Male	<100	100	<100	<100	<100	<100	<100	137	>90	<20	<20
61	CLLV61	On.Tx	Male	<100	4297	<100	<100	<100	<100	360	13105	>90	<20	<20
62	CLLV62	Tx.naïve	Male	13804	179944	3920	67311	ND	ND	ND	ND	14.6	1205	585
63	CLLV63	Tx.naïve	Male	28720	>312500	6708	89175	ND	ND	ND	ND	15.2	1297	227
64	CLLV64	On.Tx	Male	<100	<100	<100	<100	ND	ND	ND	ND	>90	<20	<20
65	CLLV65	Tx.naïve	Male	1795	17017	867	16804	ND	ND	ND	ND	>90	96	<20
66	CLLV66	On.Tx	Female	<100	<100	<100	<100	ND	ND	ND	ND	>90	<20	<20
67	CLLV67	Off.Tx.CR	Male	8617	105371	2917	40868	ND	ND	ND	ND	15.4	247	322
68	CLLV68	Tx.naïve	Male	1000	14080	118	1516	<100	1254	1990	12730	>90	<20	<20
69	CLLV69	Tx.naïve	Male	36581	>312500	7678	107963	ND	ND	ND	ND	4.2	611	772
70	CLLV70	On.Tx	Female	1676	22342	<100	386	146	3190	2059	14683	>90	<20	<20
71	CLLV71	On.Tx	Male	<100	<100	<100	<100	ND	ND	ND	ND	>90	<20	<20
72	CLLV72	Off.Tx.CR	Male	4584	115885	1999	18279	ND	ND	ND	ND	32.9	195	498
73	CLLV73	On.Tx	Female	<100	<100	<100	<100	ND	ND	ND	ND	>90	<20	<20
74	CLLV74	On.Tx	Male	188	8745	230	767	<100	2989	121	4230	>90	<20	<20
75	CLLV75	On.Tx	Male	<100	<100	<100	<100	ND	ND	ND	ND	>90	<20	<20
76	CLLV76	Tx.naïve	Female	2733	34931	744	15249	ND	ND	ND	ND	87.4	61	<20
77	CLLV77	On.Tx	Female	2128	26777	454	16099	ND	ND	ND	ND	>90	65	80
78	CLLV78	Tx.naïve	Male	2903	44698	281	451	427	798	2254	25803	>90	<20	<20
79	CLLV79	Tx.naïve	Female	3929	62799	212	654	210	6027	2898	43683	>90	<20	<20
80	CLLV80	Off.Tx.and.R/R	Female	62608	>312500	16113	>312500	ND	ND	ND	ND	7.3	2255	2604
81	CLLV81	On.Tx	Male	<100	<100	<100	<100	ND	ND	ND	ND	>90	<20	<20
82	CLLV82	Tx.naïve	Female	1509	18125	209	1313	ND	ND	ND	ND	>90	41	<20
83	CLLV83	On.Tx	Male	<100	<100	<100	<100	ND	ND	ND	ND	>90	<20	<20
84	CLLV84	Tx.naïve	Female	22404	>312500	5557	76049	ND	ND	ND	ND	33.7	548	374
85	CLLV85	Tx.naïve	Female	1384	17623	263	6252	ND	ND	ND	ND	>90	<20	<20
86	CLLV86	Tx.naïve	Female	1791	10231	<100	<100	396	9514	3186	54983	>90	<20	<20
87	CLLV87	Tx.naïve	Male	<100	<100	<100	<100	ND	ND	ND	ND	>90	<20	<20
88	CLLV88	Tx.naïve	Female	555	11537	169	1723	ND	ND	ND	ND	>90	<20	<20
89	CLLV89	Off.Tx.CR	Male	1861	16261	284	5827	ND	ND	ND	ND	>90	<20	<20
90	CLLV90	Tx.naïve	Male	10348	92902	2163	15895	ND	ND	ND	ND	30.8	101	182
91	CLLV91	Tx.naïve	Female	1371	16682	<100	<100	ND	ND	ND	ND	>90	30	21
92	CLLV92	On.Tx	Female	<100	<100	<100	<100	ND	ND	ND	ND	>90	<20	<20
93	CLLV93	Tx.naïve	Female	3695	70113	1864	18575	ND	ND	ND	ND	51.7	139	161
94	CLLV94	Tx.naïve	Male	5006	94551	1176	15881	ND	ND	ND	ND	>90	134	290
95	CLLV95	Tx.naïve	Female	564	14560	219	2901	ND	ND	ND	ND	>90	27	29

Assay sensitivity cut-off values for Spike and RBD were >100; for the D614G and Delta neutralization assays >20; and >90% for RBD/ACE2 binding.

Abbreviations: EC_{50}, half-maximal effective concentration; ID_{50}, half-maximal neutralizing titers; ND, no data; Tx, treatment; CR, clinical remission, R/R, relapsed refractory.

Table S3. Serologic responses and univariate analyses for SARS-CoV-2 vaccinated healthy controls and CLL patients by disease status, Related to Figure 1.

A.

Quantitation of serologic responses for healthy control and CLL patients by disease status

		HC	Total CLL	Tx naïve	On Tx	Off Tx CR	Off Tx and R/R
**Spike**	n	30	95	45	34	9	7
EP	Responders	30	65	43	9	9	4
	Response rate (%)	100	68.4	95.6	26.5	100	57.1
EC_{50}	Median	15355	662	2733	<100	2740	305
	IQR	27245	3595	5137.5	0	12885	4235
	95% CI of median						
	Lower confidence limit	9754	188	1000	<100	870	100
	Upper confidence limit	23979	1791	3800	<100	19885	62608
**RBD**	n	30	95	45	34	9	7
EP	Responders	30	51	35	4	9	3
	Response rate (%)	100	53.7	77.8	11.8	100	42.9
EC_{50}	Median	3530	118	263	<100	708	<100
	IQR	8376	608	1690	0	2863	66
	95% CI of median						
	Lower confidence limit	2396	100	169	<100	247	<100
	Upper confidence limit	6343	212	1172	<100	3338	16113
**D614G**	n	30	95	45	34	9	7
Neut ID_{50}	Responders	29	40	28	1	8	3
	Response rate (%)	96.7	42.1	62.2	2.9	88.9	42.9
	Median	463.9	<20	30.0	<20	65.1	<20
	IQR	995.8	81.5	249	0	347.8	647.4
	95% CI of median						
	Lower confidence limit	367.8	<20	<20	<20	21.8	<20
	Upper confidence limit	1046.0	21.8	139.5	<20	508.4	2255
**Delta**	n	30	93	45	32	9	7
Neut ID_{50}	Responders	28	35	24	1	7	3
	Response rate (%)	93.3	37.6	53.3	3.1	77.8	42.9
	Median	345.9	<20	20.5	<20	156.4	<20
	IQR	713.1	105	262.8	0	396.1	287.7
	95% CI of median						
	Lower confidence limit	170.7	<20	<20	<20	<20	<20
	Upper confidence limit	651.2	<20	123.4	<20	497.8	2604
**ACE2/RBD binding (%)**	n	30	95	45	34	9	7
	Responders	30	28	19	0	7	2
	Response rate (%)	100	29.5	42.2	0	77.8	28.6
	Median	16	>90	>90	>90	58.7	>90
	IQR	29.4	34.4	57.0	0	69.3	36.0
	95% CI of median						
	Lower confidence limit	5.5	>90	62.2	>90	15.4	7.3
	Upper confidence limit	22.3	>90	>90	>90	>90	>90

Abbreviations: EP - endpoint; EC_{50}, half-maximal effective concentration; ID_{50}, half-maximal neutralizing titers; Tx, treatment; CR, clinical remission, R/R, relapsed refractory.

B.

Univariate analysis of response rates and median titers by serologic category and CLL disease status

		Naïve vs. On Tx	Naïve vs. Off Tx CR	Naïve vs. Off Tx and R/R	On Tx vs. Off Tx CR	On Tx vs. Off Tx and R/R	Off Tx CR vs. Off Tx and R/R
**Spike**	Fisher's P value	<0.0001	>0.9999	0.0139	<0.0001	0.1807	0.0625
EC_{50}	Odds ratio	59.72	0.000	16.13	0.000	0.27	+infinity
	95% CI	12.08 to 269.4	0.000 to 11.08	2.422 to 100.5	0.000 to 0.1973	0.06102 to 1.207	1.339 to +infinity
	Dunn's P value	<0.0001	>0.9999	>0.9999	0.0001	0.3552	0.6165
**RBD**	Fisher's P value	<0.0001	0.1832	0.0745	<0.0001	0.082	0.0192
EC_{50}	Odds ratio	26.25	0.000	4.667	0.000	0.1778	+infinity
	95% CI	7.086 to 77.69	0.000 to 1.834	1.066 to 20.22	0.000 to 0.09316	0.02802 to 0.9590	1.377 to +infinity
	Dunn's P value	<0.0001	0.6814	0.5688	<0.0001	>0.9999	0.0759
**D614G**	Fisher's P value	<0.0001	0.244	0.4205	<0.0001	0.0121	0.1058
Neut ID_{50}	Odds ratio	54.35	0.206	2.196	0.004	0.0404	10.67
	95% CI	7.844 to 572.7	0.01763 to 1.327	0.5279 to 9.407	0.0003463 to 0.07701	0.003069 to 0.3710	0.7953 to 143.0
	Dunn's P value	<0.0001	>0.9999	>0.9999	0.0009	0.1699	>0.9999
**Delta**	Fisher's P value	<0.0001	0.2728	0.6983	<0.0001	0.014	0.3024
Neut ID_{50}	Odds ratio	35.43	0.327	1.524	0.009	0.04301	4.667
	95% CI	5.150 to 376.3	0.06427 to 1.493	0.3707 to 6.544	0.0008165 to 0.1287	0.003261 to 0.3953	0.5081 to 31.88
	Dunn's P value	0.0006	0.4768	>0.9999	0.0003	0.2077	>0.9999
**ACE2/RBD binding (%)**	Fisher's P value	<0.0001	0.0721	0.6872	<0.0001	0.0256	0.1262
	Odds ratio	+infinity	0.2088	1.827	0.000	0.000	8.75
	95% CI	5.855 to +infinity	0.04142 to 0.9710	0.3181 to 9.849	0.000 to 0.05514	0.000 to 0.4059	0.9391 to 61.89
	Dunn's P value	<0.0001	0.2507	>0.9999	<0.0001	>0.9999	0.0954

Dichotomous variables were calculated by Fisher's exact test. P values for differences in medians were calculated by Dunn's multiple comparisons test.

Table S4. Clinical determinants of serologic and neutralizing responses in CLL vaccinees, Related to Figures 1 & 4.

A. Categorical univariate analysis

Clinical Variable	Spike (EP)	RBD (EP)	D614G (Neut ID_{50})	Delta (Neut ID_{50})	ACE2/RBD Binding
Age ≥ 65 yo	NS	NS	NS	NS	NS
Gender	NS	NS	NS	NS	NS
Disease status	< 0.0001	< 0.0001	< 0.0001	< 0.0001	< 0.0001
Rai stage II-IV (no)	NS	0.038	0.034	NS	NS
IGHV mutation status (MT)	0.031	0.013	NS	NS	NS
CD38 >20%	NS	NS	NS	NS	NS
FISH cytogenetics	NS	NS	NS	NS	NS
β2-microglobulin ≤ 2.4	0.0015	0.023	< 0.0001	0.0094	0.013
Prior therapy (no)	< 0.0001	< 0.0001	< 0.0001	0.0030	0.013
Anti CD20 ≥ 12 months	0.0082	0.0011	0.0030	0.0075	0.018
BTK inhibitor therapy	NS	NS	NS	NS	NS
ALC < 5,000, (10^9/L)	0.047	0.014	NS	NS	NS
IVIg prophylaxis (no)	0.0011	0.019	0.010	0.052	NS
IgG ≥ 650 mg/dL	0.010	0.0027	NS	NS	NS
IgM ≥ 40 mg/dL	NS	NS	NS	NS	NS
IgA ≥ 60 mg/dL	NS	0.035	NS	NS	NS
Pfizer or Moderna vaccine	NS	NS	NS	NS	NS
Months from vaccination	NS	0.046	NS	NS	NS

Comparisons between variables were calculated by Fisher's exact test.

Assay sensitivity cut-off values for Spike and RBD were >100; for the D614G and Delta neutralization assays >20; and >90% for RBD/ACE2 binding.

Correlates with significant p values are highlighted green.

Note disease status and months from vaccination encompass multiple catagories.

Abbreviations: EP, endpoint titer; Neut ID50, half-maximal neutralizing titer; NS, not significant; IGHV, immunoglobulin heavy chain variable region gene; MT, mutated; FISH, fluorescence in situ hybridization; BTK, Bruton's tyrosine kinase; ALC, absolute lymphocyte count; IVIg, intravenous immunoglobulin; Pfizer-BioNTech, BNT162b2; Moderna, mRNA-2173.

B. Multivariate analysis

Clinical Variable	Spike (EP)			D614G (Neut ID_{50})		
	P value	OR	95% CI	P value	OR	95% CI
Age ≥ 65 yo	0.26	0.42	0.079-1.9	0.87	1.1	0.29-4.5
Male sex	0.65	1.4	0.36-5.6	0.69	1.3	0.37-4.4
Rai stage II-IV	0.63	1.8	0.14-18	0.24	3.5	0.46-56
Unmutated IGHV	0.94	0.93	0.11-6	0.83	1.2	0.28-5.1
On therapy	0.0028	62	3.6-3500	0.041	40	1.2-2500
Off therapy and R/R	0.038	7.7	1.1-64	0.6	1.7	0.25-13
Off therapy CR	0.54	3.7	0.018-250	0.81	0.71	0.034-17
ALC > 5,000, (10^9/L)	0.3	3.8	0.37-130	0.13	5.2	0.65-110
IVIg prophylaxis therapy	0.018	5.2	1.3-29	0.052	4.4	0.99-25
Pfizer vaccination	0.59	1.4	0.39-5.9	0.0056	5.8	1.6-27
2 months from vaccination	0.3	2.3	0.49-12	0.9	1.1	0.24-5
>3 months from vaccination	0.51	0.59	0.11-2.8	0.91	1.1	0.25-5

Firth logistic regression was used to examine the association of serological responses with clinical variables.

Abbreviations: R/R, relapsed refractory; CR, clinical remission.

Table S5. Subset frequencies, SARS-CoV-2 S peptide-specific AIM responses, and effector functions in healthy control and CLL donor T cells, Related to Figures 2, 5, S1 & S2.

A.

	ID	Status	Gender	Total T cells	CD4 T cell frequencies: Total	CD4 T cell frequencies: Naïve and memory subsets				CD8 T cell frequencies: Total	CD8 T cell frequencies: Naïve and memory subsets			
				CD19-CD3+	CD3+CD4+	(Tcm)Q1: CD45RA-, CCR7+	(naive)Q2: CD45RA+, CCR7+	(Terma)Q3: CD45RA+, CCR7-	(Tem)Q4: CD45RA-, CCR7-	CD3+CD8+	(Tcm)Q1: CD45RA-, CCR7+	(naive)Q2: CD45RA+, CCR7+	(Terma)Q3: CD45RA+, CCR7-	(Tem)Q4: CD45RA-, CCR7-
1	**HC2**	Healthy	Female	80.7	80.2	30.9	49.8	2.4	16.9	17.1	10	27.1	38	24.8
2	**HC5**	Healthy	Male	74.1	79.1	24.9	36.5	6.16	32.5	10.3	8	30.1	21.5	40.4
3	**HC8**	Healthy	Male	77.9	77.2	25.7	37.2	6.69	30.4	16.3	2.95	1.85	74.1	21.1
4	**HC9**	Healthy	Female	82.1	91.3	36.6	46.6	2.96	13.8	4.92	8.47	17.8	48.5	25.2
5	**HC11**	Healthy	Male	85.6	87.7	23.1	60.9	2.33	13.6	7.69	9.01	23.4	21.5	46
6	**HC13**	Healthy	Male	40.9	92.9	30.4	36.5	4.04	29.1	3.32	3.22	14.8	56.6	25.4
7	**HC14**	Healthy	Female	80.3	80.2	17.7	44.4	4.56	33.4	13.8	5.09	20.2	32.9	41.8
8	**HC15**	Healthy	Female	86.9	92.9	27.1	53.1	2.6	17.2	3.31	18.5	47.7	6.68	27.1
9	**HC16**	Healthy	Female	90.1	81.5	28.2	33.9	5.04	32.9	13.4	18.5	39.3	12.2	30
10	**HC17**	Healthy	Female	90	76.2	26.7	36.8	6.08	30.4	18.3	7.33	22.1	38.3	32.2
11	**HC18**	Healthy	Male	80.2	86	22.3	23.2	10.7	43.8	7.17	9.86	26.1	18.9	45.1
12	**HC20**	Healthy	Male	76.7	82.1	27.7	38.8	5.5	28	8.4	6.26	45.2	21.7	26.9
13	**HC21**	Healthy	Male	76.5	77.4	26.8	41.6	8.2	23.4	7.27	13.8	31.9	9.45	44.9
14	**HC22**	Healthy	Male	80.3	65.1	23.5	46.5	6.67	23.3	19.7	32.6	28.7	14.6	24.1
15	**HC23**	Healthy	Female	89.4	72.3	25.4	39.8	7.94	26.8	21.2	9.66	27.2	17.9	45.3
16	**HC24**	Healthy	Male	78.7	66.8	30.4	50.6	2.36	16.6	22.8	7.69	53	4.56	34.7
17	**HC25**	Healthy	Male	80.4	53.1	22.3	24.7	5.42	47.5	16.7	7.19	12.8	9.25	70.8
18	**HC26**	Healthy	Male	60.9	82.5	19.7	42.4	9.66	28.2	4.49	6.69	45.9	5.42	42
19	**HC28**	Healthy	Male	79.1	72.9	23.2	42.9	3.94	30	18.3	10.4	3.77	51.6	34.3
20	**HC30**	Healthy	Male	76	86.3	29.6	46.4	3.8	20.2	5.62	17.7	19.7	3.63	59
21	**HC31**	Healthy	Male	84.6	40.6	17.2	26.7	11.2	44.9	8.65	9.95	18.4	32.6	39
1	**CLLV1**	On.Tx	Male	81.1	81.4	10.4	50.5	15.4	23.7	11.8	1.99	8.55	72.5	17
2	**CLLV2**	Tx.naïve	Male	7.79	82.1	45.4	26.2	1.5	27	11.4	13.9	21	35.9	29.1
3	**CLLV6**	On.Tx	Female	46.6	75.3	37.5	16	15.9	30.6	9.12	11.1	10.7	37.5	40.7
4	**CLLV12**	Tx.naïve	Male	13.2	67.7	37.7	18.3	4.32	39.7	28.1	2.88	1.57	39.2	56.4
5	**CLLV21**	Tx.naïve	Male	33.6	68.5	19.1	25.5	15.1	40.4	16.3	2.52	2.72	59.4	35.3
6	**CLLV23**	Tx.naïve	Female	9.91	85.6	40.4	32.9	2.48	24.3	9.12	16.1	11.4	26.9	45.6
7	**CLLV27**	Tx.naïve	Female	15.1	68.1	16.2	50.9	7.62	25.3	23.2	2.03	11.7	66.5	19.7
8	**CLLV30**	Tx.naïve	Male	2.12	88	19.6	44.3	7.08	29	8.37	1.26	10.6	32.6	55.6
9	**CLLV31**	Off.Tx.and.R.R	Female	7.78	45.2	19.7	3.3	4.09	72.9	44.6	2.9	1.45	52.2	43.4
10	**CLLV33**	Tx.naïve	Male	37.9	42.4	38.7	31.7	6.67	23	37.8	1.91	0.6	71	26.5
11	**CLLV34**	Tx.naïve	Female	19.5	76.1	30.7	5.89	8.08	55.3	11.1	13.5	1.81	26.7	57.9
12	**CLLV35**	Off.Tx.CR	Female	42.7	72	19.3	48.5	5.05	27.2	20.3	5.16	45	12.3	37.5
13	**CLLV36**	Tx.naïve	Female	3.3	63.5	51.6	21.5	4.05	22.8	18.3	27.3	10.7	5.81	56.2
14	**CLLV40**	Off.Tx.and.R.R	Female	0.98	51.8	20.8	14.5	11	53.7	39.8	6.29	1	22	70.7
15	**CLLV47**	Off.Tx.CR	Female	55.3	74.3	21.3	9.52	9.27	59.9	21.2	18	6.8	16	59.2
16	**CLLV48**	Tx.naïve	Female	16	75.5	14.9	54.2	7.98	23	21.5	2.04	2.33	10	85.6
17	**CLLV49**	Off.Tx.and.R.R	Male	2.45	64.5	41.9	3.33	3.88	50.9	25.6	19	1.08	25.4	54.6
18	**CLLV50**	On.Tx	Female	6.36	54.5	17.2	15.5	6.11	61.2	37.8	3.8	3.46	28.8	63.9
19	**CLLV51**	On.Tx	Male	80.1	88.1	12.9	33.1	17.9	36.2	5.85	5.04	25.3	27.1	42.5
20	**CLLV54**	Off.Tx.and.R.R	Female	4.04	76.1	19.1	70.1	2.51	8.33	6.2	8.61	26	26.5	39
21	**CLLV55**	Tx.naïve	Male	3.78	34.3	30.8	14.5	8.49	46.2	42	11.7	7.08	8.23	72.9
22	**CLLV57**	Tx.naïve	Female	1.64	54	40.1	41.9	2.31	15.6	32.6	19.5	8.22	18.6	53.7
23	**CLLV58**	Tx.naïve	Female	8.99	75	22.7	46.9	10.5	19.8	15.1	8.42	53.2	30.6	7.81
24	**CLLV59**	Off.Tx.and.R.R	Male	2.34	88.1	29.7	39.3	3.78	27.1	7.87	11.8	24.4	12.2	51.6
25	**CLLV60**	Tx.naïve	Male	20.9	51.4	29	16.1	6.57	48.4	29.1	5.1	3.18	27	64.7
26	**CLLV61**	On.Tx	Male	49	94.5	20.6	27.7	13.5	38.2	1.47	14.6	8.21	14.9	62.3
27	**CLLV62**	Tx.naïve	Male	12.4	79.6	21	33.3	9.63	36	12.5	11.6	5.41	36.6	46.3
28	**CLLV64**	On.Tx	Male	72.2	47	26.8	33	4.8	35.4	32.1	5.4	6.73	12.8	75
29	**CLLV65**	Tx.naïve	Male	9.94	88.2	21.6	57.3	2.85	18.3	9.1	4.85	7.67	49.5	37.9
30	**CLLV69**	Tx.naïve	Male	33.4	77.6	25.2	53.9	3.53	17.4	14.9	12.4	14.5	19.8	53.3
31	**CLLV70**	On.Tx	Female	1.48	71	10.8	80.3	2.4	6.45	24.7	1.92	23.2	41.7	33.2
32	**CLLV75**	On.Tx	Male	5.32	49.9	14.6	13.6	10	61.7	42.1	6.54	1.68	14	77.8
33	**CLLV78**	Tx.naïve	Male	11.3	59.2	24.4	27.2	5.13	43.3	14.8	2.36	7.13	38.1	52.4
34	**CLLV80**	Off.Tx.and.R.R	Female	4.44	56.4	33.4	15.9	3.59	47.1	39.9	5.84	1.07	20.6	72.4
35	**CLLV84**	Tx.naïve	Female	14.4	61.9	36.8	16.9	5.55	40.8	31.7	8.26	3.79	26.9	61.1
36	**CLLV86**	Tx.naïve	Female	34.7	66	32.2	41.8	2.05	24	27.7	15.1	14	36.2	34.6

Values shown for T cell subpopulations indicate frequencies (%) as determined by flow cytometry analysis.

Abbreviations: Tx, treatment; CR, clinical remission, R/R, relapsed refractory.

B.

ID	N-specific T cell subset AIM responses — AIM responders: CD4	cTfh	CD8	SUM (N)
**HC2**	0	0	0	0
**HC5**	0	0	0	0
**HC8**	0	0	0	0
**HC9**	0	0	0	0
**HC11**	0	0	0	0
**HC13**	0	0	0	0
**HC14**	0	0	0	0
**HC15**	0	0	0	0
**HC16**	0	0	0	0
**HC17**	0	0	0	0
**HC18**	0	0	0	0
**HC20**	0	0	0	0
**HC21**	0	0	0	0
**HC22**	0	0	0	0
**HC23**	0	0	1	1
**HC24**	0	0	0	0
**HC25**	0	0	0	0
**HC26**[a]	ND	ND	ND	ND
**HC28**	0	0	0	0
**HC30**[a]	ND	ND	ND	ND
**HC31**[a]	ND	ND	ND	ND
**CLLV1**	0	0	0	0
**CLLV2**	0	0	0	0
**CLLV6**	0	0	0	0
**CLLV12**	0	0	0	0
**CLLV21**	0	0	0	0
**CLLV23**	0	0	0	0
**CLLV27**	0	0	0	0
**CLLV30**	0	0	0	0
**CLLV31**	0	0	0	0
**CLLV33**	0	0	0	0
**CLLV34**	1	0	0	1
**CLLV35**	0	0	0	0
**CLLV36**	0	0	0	0
**CLLV40**	0	0	0	0
**CLLV47**	0	0	0	0
**CLLV48**	0	0	0	0
**CLLV49**	1	0	0	1
**CLLV50**	0	0	0	0
**CLLV51**	0	0	0	0
**CLLV54**	0	0	0	0
**CLLV55**	0	0	0	0
**CLLV57**	0	0	0	0
**CLLV58**	0	0	0	0
**CLLV59**	1	0	0	1
**CLLV60**	0	0	0	0
**CLLV61**	0	0	0	0
**CLLV62**	0	0	0	0
**CLLV64**	0	0	0	0
**CLLV65**	0	0	0	0
**CLLV69**	0	0	0	0
**CLLV70**	0	0	0	0
**CLLV75**	0	0	0	0
**CLLV78**	0	0	0	0
**CLLV80**	0	0	0	0
**CLLV84**	0	0	0	0
**CLLV86**	0	0	0	0

[a]Material was not available to run the assay on these samples.

Values of "1" indicate the presence and "0" the absence of a response.

Abbreviations: N, nucleocapsid; ND, no data.

C.

ID	S-specific T cell subset AIM responses — AIM responders: CD4	cTfh	CD8	Positive (S)	SUM (S)	Response frequencies: CD4	cTfh	CD8
**HC2**	1	1	1	1	3	0.35	2.13	0.28
**HC5**	1	0	0	1	1	0.06	0.00	0.09
**HC8**	1	1	1	1	3	0.83	2.81	0.20
**HC9**	1	0	0	1	1	0.09	0.61	0.00
**HC11**	1	1	0	1	2	0.31	2.74	0.00
**HC13**	0	0	0	0	0	0.00	0.59	0.00
**HC14**	1	1	0	1	2	0.28	2.13	0.15
**HC15**	1	1	1	1	3	0.80	4.35	0.07
**HC16**	1	1	1	1	3	1.44	1.31	0.44
**HC17**	1	1	1	1	3	0.46	0.57	0.20
**HC18**	1	0	1	1	2	0.49	0.18	0.90
**HC20**	1	1	0	1	2	0.24	0.13	0.03
**HC21**	1	1	1	1	3	0.22	0.34	0.51
**HC22**	1	1	1	1	3	0.26	0.43	0.17
**HC23**	1	1	1	1	3	1.83	3.01	0.19
**HC24**	1	1	0	1	2	0.34	0.54	0.11
**HC25**	1	1	0	1	2	0.81	0.98	0.09
**HC26**	1	1	0	1	2	0.52	1.38	0.00
**HC28**	0	1	0	1	1	0.10	1.64	0.30
**HC30**	0	1	1	1	2	0.28	0.78	0.14
**HC31**	0	0	0	0	0	0.14	0.41	0.01
**CLLV1**	1	1	1	1	3	0.135	2.040	0.401
**CLLV2**	0	0	0	0	0	0.000	0.000	0.000
**CLLV6**	0	0	0	0	0	0.010	0.000	0.000
**CLLV12**	1	0	0	1	1	0.360	0.038	0.023
**CLLV21**	0	0	0	0	0	0.000	0.000	0.000
**CLLV23**	0	0	0	0	0	0.030	0.000	0.160
**CLLV27**	0	0	0	0	0	0.015	0.000	0.008
**CLLV30**	0	0	0	0	0	0.000	0.000	0.000
**CLLV31**	0	0	0	0	0	0.000	0.000	0.000
**CLLV33**	0	0	0	0	0	0.021	0.000	0.051
**CLLV34**	0	0	0	0	0	0.000	0.000	0.090
**CLLV35**	0	0	0	0	0	0.031	0.000	0.059
**CLLV36**	0	0	0	0	0	0.000	0.000	0.500
**CLLV40**	0	0	0	0	0	0.000	0.000	0.110
**CLLV47**	0	0	0	0	0	0.000	0.140	0.038
**CLLV48**	0	0	0	0	0	0.061	0.680	0.000
**CLLV49**	1	0	0	1	1	0.150	1.230	0.180
**CLLV50**	0	0	0	0	0	0.000	0.000	0.000
**CLLV51**	0	0	0	0	0	0.038	0.920	0.000
**CLLV54**	0	0	0	0	0	0.065	0.000	0.000
**CLLV55**	1	1	0	1	2	0.554	2.600	0.079
**CLLV57**	0	0	0	0	0	0.035	0.240	0.000
**CLLV58**	1	1	1	1	3	0.349	2.170	0.352
**CLLV59**	1	0	0	1	1	0.092	0.930	0.053
**CLLV60**	0	0	0	0	0	0.000	0.000	0.050
**CLLV61**	0	0	0	0	0	0.039	0.110	0.000
**CLLV62**	0	0	0	0	0	0.430	0.000	0.042
**CLLV64**	1	0	0	1	1	0.140	0.000	0.033
**CLLV65**	0	0	0	0	0	0.006	0.060	0.000
**CLLV69**	1	1	0	1	2	0.720	2.240	0.060
**CLLV70**	0	0	0	0	0	0.040	2.820	0.000
**CLLV75**	0	0	1	1	1	0.069	0.130	0.085
**CLLV78**	1	1	0	1	2	0.456	1.960	0.106
**CLLV80**	0	0	0	0	0	0.030	0.000	0.009
**CLLV84**	1	1	0	1	2	0.139	0.390	0.000
**CLLV86**	0	0	1	1	1	0.170	0.820	0.173

Values of "1" indicate the presence and "0" the absence of a response.

Values shown for T cell subpopulation responses indicate frequencies (%) as determined by flow cytometry analysis.

Abbreviation: S, spike.

D.

S-specific T cell subset effector functions of healthy control and CLL vaccinees

ID	ICS T cell responders: CD4	CD8	Positive (S)	SUM (S)	CD4 T cells: IFNγ	TNFα	IL2	Grz-B	Perforin	PFS	CD8 T cells: IFNγ	TNFα	IL2	Grz-B	Perforin	PFS
**HC2**	1	1	1	2	0.021	0.064	0.070	0.000	0.002	0.241	0.135	0.016	0.073	0.122	0.170	0.197
**HC5**	1	0	1	1	0.031	0.065	0.069	0.000	0.000	0.233	0.080	0.020	0.035	0.019	0.000	0.157
**HC8**	1	1	1	2	0.007	0.017	0.119	0.001	0.003	0.241	0.000	0.000	0.165	0.000	0.000	0.121
**HC9**	1	0	1	1	0.010	0.063	0.021	0.002	0.002	0.176	0.090	0.000	0.000	0.020	0.000	0.161
**HC11**	1	1	1	2	0.019	0.031	0.032	0.000	0.003	0.239	0.230	0.119	0.000	0.000	0.036	0.182
**HC13**																
**HC14**[a]	ND	ND	ND	ND	ND	ND	ND	ND	ND	ND	ND	ND	ND	ND	ND	ND
**HC15**	1	1	1	2	0.011	0.050	0.101	0.000	0.000	0.272	0.140	0.036	0.123	0.005	0.013	0.234
**HC16**	1	1	1	2	0.015	0.086	0.129	0.000	0.000	0.250	0.156	0.097	0.123	0.004	0.013	0.293
**HC17**	1	1	1	2	0.059	0.162	0.090	0.000	0.000	0.237	0.202	0.085	0.000	0.010	0.024	0.264
**HC18**	1	1	1	2	0.102	0.210	0.190	0.000	0.006	0.274	0.182	0.072	0.000	0.029	0.020	0.244
**HC20**	1	1	1	2	0.263	0.032	0.295	0.000	0.000	0.342	0.295	0.000	0.381	0.003	0.008	0.242
**HC21**	1	1	1	2	0.147	0.164	0.136	0.000	0.001	0.308	0.273	0.019	0.000	0.053	0.113	0.262
**HC22**	1	1	1	2	0.065	0.069	0.054	0.000	0.002	0.329	0.148	0.030	0.061	0.000	0.000	0.187
**HC23**																
**HC24**	1	1	1	2	0.064	0.068	0.079	0.001	0.002	0.360	0.069	0.016	0.115	0.008	0.008	0.242
**HC25**	1	1	1	2	0.025	0.063	0.037	0.000	0.000	0.190	0.116	0.007	0.000	0.000	0.000	0.233
**HC26**[a]	ND	ND	ND	ND	ND	ND	ND	ND	ND	ND	ND	ND	ND	ND	ND	ND
**HC28**	1	1	1	2	0.000	0.006	0.138	0.000	0.000	0.172	0.090	0.030	0.174	0.046	0.050	0.177
**HC30**[a]	ND	ND	ND	ND	ND	ND	ND	ND	ND	ND	ND	ND	ND	ND	ND	ND
**HC31**																
**CLLV1**	1	1	1	2	0.000	0.021	0.462	0.000	0.000	0.216	0.124	0.237	0.097	0.006	0.009	0.303
**CLLV2**																
**CLLV6**																
**CLLV12**	0	0	0	0	0.000	0.040	0.041	0.000	0.000	0.132	0.015	0.002	0.034	0.028	0.013	0.071
**CLLV21**																
**CLLV23**																
**CLLV27**																
**CLLV30**																
**CLLV31**																
**CLLV33**																
**CLLV34**																
**CLLV35**																
**CLLV36**																
**CLLV40**																
**CLLV47**																
**CLLV48**																
**CLLV49**																
**CLLV50**																
**CLLV51**																
**CLLV54**																
**CLLV55**	0	1	1	1	0.130	1.300	0.400	0.000	0.031	0.176	0.570	0.440	0.000	0.000	0.046	0.198
**CLLV57**																
**CLLV58**	0	0	0	0	0.000	0.110	0.064	0.000	0.000	0.052	0.000	0.000	0.000	0.000	0.000	0.000
**CLLV59**																
**CLLV60**																
**CLLV61**																
**CLLV62**																
**CLLV64**	0	0	0	0	0.000	0.033	0.000	0.050	0.150	0.000	0.000	0.470	0.000	0.040	0.030	0.001
**CLLV65**																
**CLLV69**[a]	ND	ND	ND	ND	ND	ND	ND	ND	ND	ND	ND	ND	ND	ND	ND	ND
**CLLV70**																
**CLLV75**[a]	ND	ND	ND	ND	ND	ND	ND	ND	ND	ND	ND	ND	ND	ND	ND	ND
**CLLV78**[a]	ND	ND	ND	ND	ND	ND	ND	ND	ND	ND	ND	ND	ND	ND	ND	ND
**CLLV80**																
**CLLV84**	0	1	1	1	0.002	0.048	0.000	0.002	0.000	0.166	0.047	0.004	0.000	0.010	0.013	0.182
**CLLV86**	1	1	1	2	0.019	0.120	0.050	0.000	0.000	0.167	0.046	0.020	0.044	0.021	0.008	0.204

[a]Material was not available to run the assay on these samples.

Values of "1" indicate the presence and "0" the absence of a response. For values left blank data are not applicable.

Values shown for T cell subpopulation responses indicate frequencies (%) as determined by flow cytometry analysis.

Abbreviations: ICS, intracellular cytokine staining; PFS, polyfunctionality score calculated by COMPASS; ND, no data.
